# Soil and vegetation water content identify the main terrestrial ecosystem changes

**DOI:** 10.1093/nsr/nwad026

**Published:** 2023-02-07

**Authors:** Diego Bueso, Maria Piles, Philippe Ciais, Jean-Pierre Wigneron, Álvaro Moreno-Martínez, Gustau Camps-Valls

**Affiliations:** Image Processing Laboratory, Universitat de València, Valencia 46010, Spain; Image Processing Laboratory, Universitat de València, Valencia 46010, Spain; Laboratoire des Sciences du Climat et de l’Environnement Université Paris Saclay, Gif-sur-Yvette 91190, France; INRA Centre de Bordeaux Aquitaine, Villenave 33140, France; Image Processing Laboratory, Universitat de València, Valencia 46010, Spain; Image Processing Laboratory, Universitat de València, Valencia 46010, Spain

**Keywords:** soil moisture, vegetation water content, ecosystem, trend, remote sensing, soil moisture and ocean salinity satellite

## Abstract

Environmental change is a consequence of many interrelated factors. How vegetation responds to natural and human activity still needs to be well established, quantified and understood. Recent satellite missions providing hydrologic and ecological indicators enable better monitoring of Earth system changes, yet there is no automatic way to address this issue directly from observations. Here, we develop an observation-based methodology to capture evidence of changes in global terrestrial ecosystems and attribute these changes to natural or anthropogenic activity. We use the longest time record of global microwave L-band soil moisture and vegetation optical depth as satellite data and build spatially explicit maps of change in soil and vegetation water content and biomass reflecting large ecosystem changes during the last decade, 2010–20. Regions of prominent trends (from $-8\%$ to 9% per year) are observed, especially in humid and semi-arid climates. We further combine such trends with land cover change maps, vegetation greenness and precipitation variability to assess their relationship with major documented ecosystem changes. Several regions emerge from our results. They cluster changes according to human activity drivers, including deforestation (Amazon, Central Africa) and wildfires (East Australia), artificial reforestation (South-East China), abandonment of farm fields (Central Russia) and climate shifts related to changes in precipitation variability (East Africa, North America and Central Argentina). Using the high sensitivity of soil and vegetation water content to ecosystem changes, microwave satellite observations enable us to quantify and attribute global vegetation responses to climate or anthropogenic activities as a direct measure of environmental changes and the mechanisms driving them.

## Teaser

This work identifies and quantifies the main large ecosystem changes during 2010-2020. Regions with prominent joint SM and VOD trends stand out, and can be semantically attributed to climate or anthropogenic activities, from reforestation and deforestation to irrigation changes and wildfires.

## INTRODUCTION

The ‘dry gets drier, wet gets wetter’ paradigm was suggested to describe the evident effects of climate warming in most terrestrial ecosystems [1–3]. Land water availability is a crucial resource for vegetation development and health status, as the limitation of freshwater can induce permanent changes in ecosystems. Coupling between soil and vegetation water content is key to understanding ecological dynamics in large ecosystems and their interaction with climate and human activities [4,5]. Plants play a crucial role in mediating the interactions between the land and the atmosphere. However, it is unclear how these interactions might shift with a changing climate, such as drier air, increased temperatures or changing rainfall patterns. In addition, human activity increasingly threatens the viability and resilience of ecosystems and the human societies that depend upon them. The effects of these threats can be profound and, in recent years, have become increasingly observable [2]. Nevertheless, how can one identify and disentangle natural and anthropogenic drivers of vegetation change globally, objectively and solely from observational data?

Identifying dominant drivers of change and their interactions with other stressors is problematic partly because not all ecological variables can be measured with comparable accuracy. Note that they are only typically available over short periods and are subject to noise, making their sensitivity harder to assess. Also, trends are difficult to assess since they depend on the specific space and time scale considered and the interactions between drivers. Climate shifts and human activities can cause large ecosystem changes at global and interannual scales. Differentiating between the two is a very challenging yet needed endeavour. Changes in air temperature and precipitation can limit vegetation growth and induce changes in large ecosystems [6]. On the other hand, human activity directly impacts the Earth’s vegetation through forest management, such as deforestation, reforestation and cultivation abandonment. Humans consume large amounts of resources for their own needs. Some examples include mining natural resources like coal, human appropriation of net primary production and biomass, and clearing forests for urbanisation and wood use [7]. Deforestation and forest degradation can happen quickly, such as when a forest is clear-cut to make way for a palm oil plantation or a new settlement [8]. Nevertheless, this may also happen gradually due to ongoing forest degradation as temperatures rise due to climate change and in response to selective logging and fragmentation of forests [9–11].

Identifying the different causes that are driving ecosystem functioning is a non-trivial problem on a global scale. In particular, there is a need to understand the ecological dynamics of these climate impacts, identify hotspots of vulnerability and resilience and identify management interventions that may assist biosphere resilience to climate change. Some studies relying on satellite data have detected a global increase in vegetation greenness [12–14]. The attribution of these changes is usually reported as climate change consequences dominated by land cover changes of different origins. A review can be found in [15]. Regionally, these trends can specifically be attributed to changes in precipitation [16,17], an increase in water vapour pressure deficit due to a rise in air temperature [18,19] or land management change [1].

Studies typically use the leaf area index, the normalised difference vegetation index (NDVI) or the gross primary production from satellite observations as proxies for vegetation changes [12–14,20]. All these studies have systematically used one index to identify major changes and their drivers. However, some recent works have introduced the analysis of combined indices to study greening with satellite-derived global vegetation indices, and solar-induced fluorescence (SIF) [15] to study asynchrony in vegetation phenology between NDVI, vegetation optical depth (VOD) and SIF [21], or for crop yield monitoring with the combination of vegetation indices, soil moisture (SM) and meteorological drivers [22].

In this work, we alternatively posit that the combined analysis of soil and vegetation water content is more sensitive to changes in ecosystem functioning and can jointly disentangle the different natural and anthropogenic causes of change in global terrestrial ecosystems. We use soil moisture and vegetation optical depth estimates derived from passive microwave remote sensing sensors to detect large ecosystems sensitive to water content changes and attribute the causes. In particular, we use the Soil Moisture and Ocean Salinity (SMOS) satellite, which provides the longest record of L-band-based SM and VOD retrievals. VOD indicates vegetation water content and aboveground biomass since it captures the extinction effects of the microwave radiation propagating in the vegetation canopy [23,24]. Both SM and VOD are valuable hydrologic and ecological indicators, important for a breadth of scientific studies and applications such as biomass estimation [25–27], crop yield assessment [22,28], agricultural drought monitoring [29,30] and analysis of water exchange in the soil-plant-atmosphere system [5,31–33]. Here, we investigate the interannual trends of SM and VOD at a global scale to identify the distinct factors driving ecosystems out of balance in recent times. We use the Mann-Kendall trend test (see the Methods section) with a spatio-temporal approach to finding significant clusters with concurrent monotonic changes in SM and VOD over the last decade (2010–22); see the Data section. We cross-validate results by comparing the extracted trends with precipitation records and vegetation greenness data (NDVI; see the online supplementary material). Different spatially homogeneous groups emerge, allowing us to identify causes/drivers of monotonic changes in five semantically compact regions using only satellite-based estimates of soil and vegetation water content.

## RESULTS

**Figure 1.**
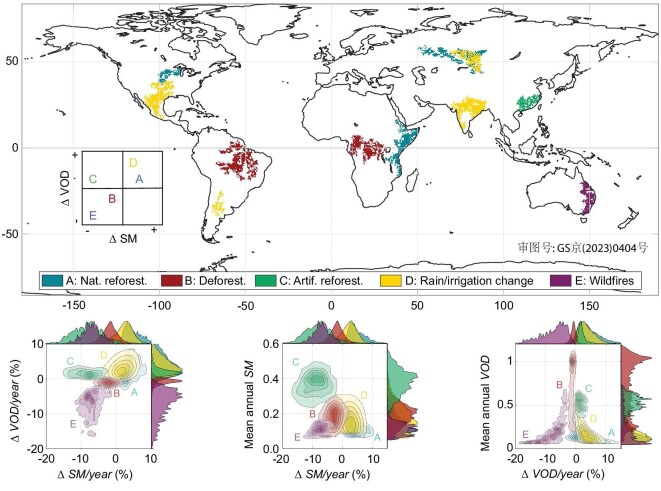
Top: the five identified clusters of SM and VOD trends globally, with their semantic attribution. Bottom: identified clusters represented in bivariate spaces between (left) trends of SM and VOD, (middle) trends in SM and mean annual SM, and (right) trends of VOD and mean annual VOD. Each cluster emerges as an independent group from the joint SM and VOD trend space: (bottom left) clusters A and D show a positive change in both SM and VOD, clusters B and E show negative SM and VOD changes (more pronounced in E), cluster C shows a negative SM change and a positive VOD change. Estimated joint and marginal distributions for SM and VOD highlight the distinct monotonic trends of soil and vegetation water content in each cluster. The relation between the relative trend in VOD to its annual mean (right) shows how regions with low VOD levels, on average, are more sensitive to VOD changes. Using the SM trends alone (middle) allows us to identify all clusters but C, an exception due to artificial reforestation and the induced need for groundwater. Clusters A and D become separable using the trends in VOD and rainfall, which are more pronounced in D. See Fig. S3 within the online supplementary materials.

Soil and water are dominant natural resources for plant growth. Soil provides the mechanical support and nutrient reservoir necessary for plant development, while water is essential for plant life processes. The water exchange in the soil-plant-atmosphere continuum depends on the species and their properties. However, it is also conditioned on large-scale features such as the dominant land cover type, the soil properties and the climate region. Exploring these dependencies is key to understanding the main drivers of ecosystem changes. Owing to the high sensitivity of SM and VOD from microwave L-band satellites to water content variability, they are used here to quantify and attribute large ecosystem changes to climate or human activities for the last decade (2010–20). We extracted monotonic trends of SM and VOD separately by a robust algorithm [13]; see the Methods section for details. This automatic approach detected significant changes in SM and VOD, yielding five homogeneous spatial groups globally; see Fig. 1. Each cluster depicts a monotonic change in SM and VOD yearly. Interestingly, these main large ecosystem changes can only be identified by considering the joint distribution of SM and VOD trends (not the marginals). See the lower plots in Fig. 1. Actually, better separability cluster scores are achieved with SM and VOD than with SM or VOD alone, or with pairwise combinations with NDVI (see Sec. S3 within the online supplementary material). The distinct clusters can be attributed to five main classes related to natural and anthropogenic activities and have been reported independently in the literature (Table 1).

**Table 1.** Clusters and area of each identified region in Fig. 1; semantic classification and attribution of the clusters according to the literature.

Cluster	Regions	Area (10^3^ · km^2^)	Classification	References
A	Russia	1338	Natural	[34,35]
	North America	385	reforestation	[36–38]
	East Africa	1461		[17,39–41]
B	Amazon	1698	Deforestation	[42–46]
	Central Africa	1144		[47,48]
C	Southeast China	469	Artificial	[49–51]
			reforestation	
D	India	1103	Rain shift or	[1,52–56]
	Argentina	269	irrigation change	[35,38,57]
	Russia	631		[34,35]
	Central North America	1117		[36–38]
E	East Australia	657	Wildfires	[58–62]

**Figure 2.**
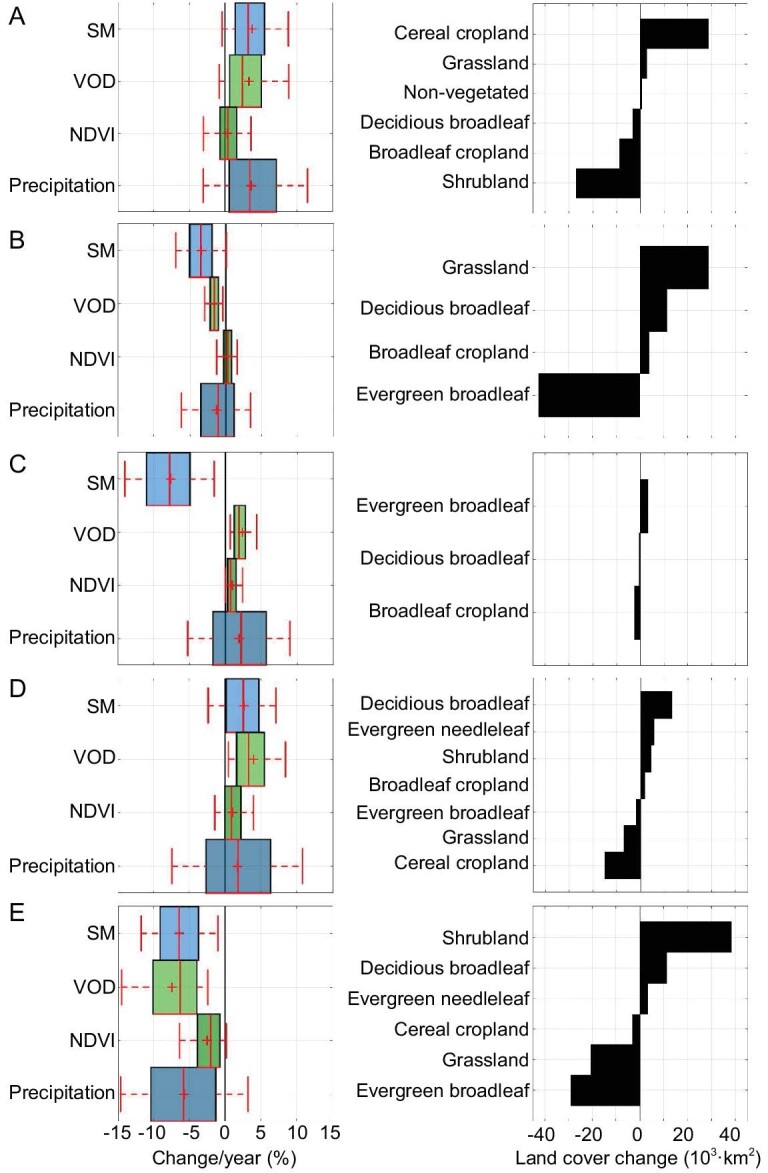
Boxplots of annual trends (in percentages) of SM, VOD, NDVI and accumulated precipitation (left column) and land cover change (right column) for the period 2010–20 for clusters A to E (cf. Fig. 1 and Table 1). Land cover changes and trends unmask the major changes in the identified clusters. Cluster A is related to natural reforestation and shows increasing trends in SM and precipitation followed by an increase in VOD. This reflects increased water supply and plant growth, mainly related to cereal croplands. Cluster B is related to deforestation and rainfall change and reflects a decreasing trend in all four variables. It appears more pronounced in SM and VOD, and the degradation of forested areas transitioning to grasslands. Cluster C, related to artificial reforestation, shows an increase in VOD and a notable decrease in SM, probably related to the increasing water demand of new forested areas. The fact that the NDVI changes are less pronounced can be due to the saturation effect of optical indices in high biomass regions, which does not affect VOD in the L-band. Cluster D, related to rain shift or irrigation changes, shows a notable increase in VOD, followed by SM and precipitation, which correspond to two major land-use changes depending on the region: either expansion of broadleaf croplands (e.g. northern India peninsula) or encroachment of grassland becoming forest (e.g. Central North America). Cluster E, related to wildfires and land clearance, shows a notable decrease in all four variables and conversion of forest and grasslands into shrublands.

Clusters A and D show a positive change in both SM and VOD, clusters B and E show negative SM and VOD changes (more pronounced in E), and cluster C shows a negative SM change and a positive VOD change. Five regions emerged with positive SM change, placed in Russia, African East Coast, Indian Peninsula, Argentina and North America (A and D in Fig. 1). This group of positive SM and VOD change can be split into two by observing their joint trend in annual cumulative precipitation, with cluster A exhibiting a bigger change in rainfall (see Fig. S3 within the online supplementary material). Focusing on the clusters with negative SM and VOD change, we found three regions in Amazonia, Central Africa and the East Coast of Australia (B and E in Fig. 1). Here, the Australian region differentiates from the tropical forest areas in Amazonia and Central Africa due to its stronger SM and VOD negative trends over the last decade. A special case is found in South-East China (C in Fig. 1), where a positive change in VOD but negative SM with no major change in rainfall is observed (see Fig. 2C). No clusters were centred between the axis of negative VOD and positive SM changes except for cluster A, where VOD change is spatially heterogeneous, presenting mainly positive but also negative changes.

The positive change in SM and VOD observed in the African East Coast, Central Russia and North America results from natural herbaceous encroachment (Africa) and woody-plant encroachment (Russia and North America) (Fig. 1, cluster A). The Eastern Africa region is mainly arid and semi-arid, where vegetation growth is strongly water limited; thus, an increase in rainfall is followed by an increase in biomass, which is observed as an increase in SM and VOD (Fig. 2D) [17,39–41]. The Central Russia region is characterised by the abandonment of farm fields after the fall of the Soviet Union, which led to an increase in biomass [34,35]. Here, vegetation is not water limited, yet a parallel increase in SM and VOD is also observed; cf. Fig. 2A. The Central North American pattern can be explained by a combined increase in annual cumulative rainfall, farmland use and irrigation [38]. The increase in irrigation in this area is probably linked to an increase in groundwater [63].

**Figure 3.**
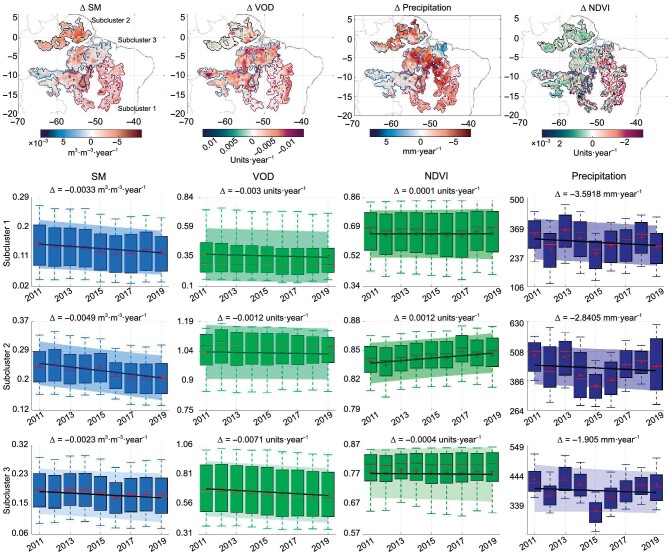
Trend analysis over the three identified subclusters in the Amazon rainforest of cluster B: subcluster 1 (southern region), subcluster 2 (northern region), subcluster 3 (middle region). Top: spatial distribution of changes over 2010–20 for the subclusters with distinct trends. The dotted pixels represent a monotonic change with a *p*-value <0.01. Bottom: annual boxplots of SM, VOD, NDVI and precipitation in each subcluster with its estimated mean linear trend (central line) and standard deviation (shaded area). The three subclusters have in common the monotonic decrease in SM and accumulated precipitation but differ in the VOD trend. NDVI is insensitive to major changes. Precipitation interannual changes are mostly related to climate variability.

The negative change in SM and VOD observed in the Amazon, Central Africa and (to a greater extent) in eastern Australia (Fig. 1, clusters B and E) corresponds to deforestation and wildfires and match with previous uncored global deforested regions [64]. Australia shows a stronger decrease in SM and VOD over the last decade, which may be related to the rapid degradation of forest areas, probably caused by an increase in wildfires due to climate change and Australia’s government agreement in 2009 to legalise the land clearing to extract wood [35,59]. The Central Africa region does not show a significant change in SM but a clear monotonic decrease in VOD. This can be explained by deforestation, which is plausible given the wildfire and shifting to cultivated areas by smallholders reported in the literature [47,48,65]. The observed pattern of deforestation in the Amazon can be further separated into three subclusters. The northern region is sensitive to dry conditions that follow a monotonic decrease in SM but not in VOD, where a decrease in rainfall frequency has been reported [45]. The middle and southern regions present a heavy decrease in VOD levels followed by a similar decrease in SM, where the middle region is probably related to deforestation and the southern region to the land-use change [42–44]. These patterns are further discussed in Fig. 3 below.

Clusters that emerged as a positive change in SM and VOD in the Indian peninsula, Central Argentina and Central North America correspond to major land-use changes (Fig. 1, cluster D). The India cluster is noted to have an annual variation in VOD but not in SM; see Fig. 2D. This pattern was previously reported as an increase in croplands, mostly over the northern area of the peninsula, where there is no significant change in annual rainfall but a significant change in irrigation management [52,54–66] (see Fig. S4 within the online supplementary material). A similar pattern is found in Central Argentina, where an increase in cropland and farm area over the last decade has also been reported [57]. The Central North American pattern can be explained by a combined decrease in livestock production followed by the encroachment of grassland becoming forest [38].

As shown in Fig. 1, there is a pattern related to an increase in VOD, but a decrease in SM placed in southeast China (cluster C). This region presents non-significant changes in annual rainfall over the last decade (see Fig. 2C) that appear to be unrelated to deforestation or climate shifts. An intense reforestation practice that has been reported in this region, impulsed by China’s government, in particular, to fight back the soil loss [49–51]. Furthermore, reported rural depopulation in this region has induced a decrease in harvested area and an increase in tree cover [67]. Planting trees in bare soils is probably causing a soil drying trend due to the increasing demand for water availability from new forested areas. We should note the modest increase in VOD and NDVI though in Fig. 2C, where the increase in NDVI looks smaller. This could be attributed to the fact that most of the reforestation in southeast China replaced grasslands and broadleaf croplands with evergreen broadleaf forests [49]. The sensitivity of VOD to this class cover change is (slightly) higher than that of NDVI, which saturates in densely vegetated areas, while L-band VOD does not.

Our results show that the high capabilities of L-band microwave sensors to monitor SM and VOD allow us to recover information on major ecosystem changes related to human and climate changes. Furthermore, the maps extracted from L-band VOD also reveal interesting results about the sensitivity of different regions. As shown in Fig. 1 (bottom right), semi-arid regions are found to be more sensitive to changes than high biomass regions, though.

### On the evolution of change in reforestation, deforestation and abandonment

Defining and quantifying the properties of environmental changes require defining the appropriate space and time of variation. The identified clusters are spatially homogeneous, and the associated grid cells showed an interannual monotonic change in SM and/or VOD over the last decade with the same sign of change per region. Homogeneous spatial distribution of interannual trends follows the spatio-temporal relation inside each cluster. Looking for strict monotonic significant changes over time allowed us to uncover a set of spatial clusters with a well-differentiated change precursor (Fig. 1, Table 1). Now, we turn to characterising the time evolution of the different clusters to extract important information related to the nature of the changes (Fig. 2).

Spatio-temporal covariation between regions can explain common causes. Note that, by definition, all of them are monotonic changes. Nevertheless, a difference between fast and slow time changes can be noted and used to differentiate the causes, which could have anthropogenic (e.g., deforestation/reforestation, abandonment, farmland growth) and natural (e.g., wildfires, rain shift) origins. Also, severe droughts or wet periods at the beginning of the observation record can induce an observed perturbation of vegetation. Trends in soil moisture and vegetation water content over land ecosystems are related to changes in rainfall patterns and vegetation cover. To compare the changes in the observed variables, we use rainfall and vegetation data as proxies (see the Data section). Changes in rainfall patterns are typically reflected by intensity, frequency or seasonality, but cumulative precipitation (CumPrecip) is often a better proxy to define a bioclimatic region. We use the NDVI, which is a proxy of greenness and canopy structure, as a vegetation density parameter like the leaf area index and fraction cover. Furthermore, changes in land cover, such as farmlands or deforestation, reflect the changes produced in the ecosystems. To compare the observed patterns and discriminate between climate and land cover changes, we used a global land cover map and extracted its changes over the studied decade.

Figure 2 summarises the changes in each cluster related to the sensitivity of SM, VOD, NDVI and annual CumPrecip over the last decade 2010–20, together with the land cover changes between the year 2020 and the year 2010. Let us now review the general attributions in what follows. Figure 2A shows a positive change in annual cumulative rainfall, soil moisture and vegetation water content. Land cover changes are mostly related to an increase in cereal cropland and a reduction in shrubland. Figure 2B, which has been identified as a deforestation pattern, shows a significant reduction in vegetation water content, followed by a high loss of evergreen broadleaf forest area, with a decrease in soil moisture due to climate trends [68]. Figure 2C, located in southeast China, shows a decline in soil moisture followed by an increase in the vegetation water content, being insensitive to NDVI and the annual CumPrecip. Changes in land cover are low compared to the rest of the clusters. Figure 2D, related to land-use change, shows a positive change in soil and vegetation water content and NDVI but not in the cumulative rainfall. Land cover change is diverse and mostly represents the abandonment of cereal crop fields eventually converted into forests. Figure 2E, located on the Eastern Coast of Australia, is related to heavy deforestation and wildfires. Annual changes in soil and vegetation water content, NDVI and cumulative rainfall are strongly negative, reflected in the land cover where there is a high loss of evergreen forest areas becoming shrublands.

Because of the change in climatic variability, rainfall patterns impact soil moisture in the short term. However, they have a long-term lagged effect on vegetation, shorter in grassland and shrubland and longer in forests. As can be seen in Fig. 2A, cluster A reveals a positive change in soil moisture and annual cumulative rainfall. This change has a different response for each region in the cluster. The North American region is mostly located in the corn belt, which accounts for major changes in the cereal cropland area with an increase in area and positive change in annual cumulative rainfall [38]. The Eastern Africa region is mostly related to changes in the seasonality of rain and the herbaceous ecosystems’ short-term response, making spring rainfall more intense and less frequent [39]. Here, we can note the high temporal correlation (R^2^ = 0.83) between soil and vegetation water content due to the interannual variability of the rain and the short response of both variables [39]. The Russian region is an extension of old abandoned farmlands due to the collapse of the Soviet Union [34]. This region is distinct from natural reforestation and shows a positive response to an increase in annual cumulative rainfall [35]. In the three analysed regions, rainfall patterns changed and produced soil moisture and vegetation changes, reflecting the loss of shrublands in the last decade.

### Identifying and quantifying distinct deforestation subclusters

The regions of the Amazon and Central African rainforests are largely reported as areas suffering continuous deforestation due to human activity as land clearing for primary matter extraction and expansion of pasture and crop fields [43,45–48]. As a result of forest degradation, large areas of the evergreen forest became grassland (see Fig. 2B).

In the case of Central Africa, soil and vegetation water content decreased in differentiated areas, with the eastern region being drier (lower SM) and the western region more related to vegetation change (lower VOD). Rain patterns do not change significantly in this region during the study period, and the NDVI is not particularly sensitive to changes in vegetation. The estimated deforested area in Central Africa here is 11 000 km^2^ in front of the 22 000 km^2^ reported in the bibliography [48]. This decadal underestimation could probably be due to the coarse spatial resolution used here, which cannot detect small changes in local scales yet is enough to identify changes in continental scales [69].

The Amazon rainforest suffered continuous deforestation and loss of evergreen forest area over the last decade. The temporal evolution of degradation is steady over the studied period, and the spatial distribution of rainfall degradation is equal across the region. In the detection of monotonic changes, we identify three subclusters with distinct annual trends (see Fig. 3), which represent the different spatial distributions of the estimated joint changes in soil and vegetation water content. A decrease in soil moisture is generally detected in the three subclusters, which is spatially correlated with the changes in rainfall patterns (mean decrease of 0.0053 of m^3^ ·m^−3^ per year and 4.7 mm per year, respectively). The temporal evolution of cumulative rainfall also shows the effect of the strong El Niño event of 2015–6, which induced drought over the Amazon as reflected by the soil moisture evolution. In subcluster 2 (northern region), the annual accumulated rainfall shows a continuous decrease followed by a strong soil moisture decrease [68,70–72]. In [43], a trend analysis of aboveground biomass (AGB) during 2010–9 using L-band VOD of the Amazon region was presented. The AGB losses were attributed to deforestation and degradation, probably due to the recent withdrawal of forest protection policies. The spatial patterns generally match ours.

Changes in vegetation water content are primarily negative in subcluster 3 (middle region), clearly characterised by a steady and strong decrease. This subcluster correlates spatially with reported deforestation areas during the studied decade [43]. It contains the major land cover changes related to the loss of evergreen forests. They correspond to areas with a high decrease in vegetation water content. The estimated area of deforestation here is 36 000 km^2^ compared to 42 000 km^2^ reported in the bibliography [46]. This shows that soil moisture in the Amazon rainforest is sensitive to changes in rainfall patterns. However, vegetation water content is sensitive to rainforest loss and biomass loss. We note that, as in the case of Central Africa, the NDVI is not sensitive to large biomass changes in rainforest areas, unlike SM (subcluster 2) and VOD (subcluster 3). See Sec. S5 within the online supplementary material for a discussion on the SMOS-IC L-VOD product and its sensitivity as proxies to biomass.

### Global hotspots of reforestation, abandonment and deforestation

Artificial reforestation is called active reforestation when it involves human action by planting trees or facilitating the conditions to correct forest development. China is an example of how human action can change large areas of degraded vegetation by planting trees to restore forest areas [49–51]. The identified cluster C in southeast China shows a significant positive increase in vegetation water content ($+4.3\%$ per year) and NDVI ($+2.5\%$ per year), and a strong decrease in soil moisture ($-10.6\%$ per year) with no significant changes in NDVI and annual accumulated rainfall. It has been reported that L-band VOD is almost insensitive to saturation effects even in dense forest canopies, unlike optical indices like NDVI (cf. Sec. S5 within the online supplementary material and [73–75]). Land cover change reveals a low change from cropland to forest areas (6000 km^2^), which results from the reforestation that started in the 2000s [1,51]. An increase in water content and a decrease in soil moisture is interpreted as an extra drain of soil water due to the new trees in a new forested area without an unbalanced water cycle and the water stress induced by the younger forest.

Human activity has an impact on the ecosystem and so on vegetation cover. Besides, the degradation of vegetated areas occurs due to increased human activities such as natural resource extraction or expansion of farmlands and croplands. Contraction of human activities such as agricultural intensification also impacts the ecosystem, allowing the restoration of forest lands. Cluster D, referred to in Fig. 1, Table 1 and Fig. 2D, has been identified as a result of a reduction in human activities due to the abandonment of farm fields and croplands. The identified regions cover parts of North America, Argentina and Russia that present an increase in soil and vegetation water content followed by an increase in deciduous broadleaf forests and a reduction of grassland areas (Fig. 2D). Literature has reported changes in these regions due to population migration to urbanised areas as in the case of North America and Argentina [38,57] and the collapse of the Soviet Union in Russia [34,35]. The increase in vegetation water content was, on average, 3.9% per year. Also, its time evolution is highly correlated with the soil moisture changes, increasing at a ratio of 2.7% per year. Another region is identified in the same cluster located in the Indian peninsula, showing a different vegetation land cover evolution. Identified areas are insensitive to changes in soil moisture, NDVI and annual cumulated rainfall, but have a high increase in vegetation water content (5.1% per year on average). Land cover change reveals a loss of 8000 km^2^ of cereal cropland becoming broadleaf cropland, and shrubland and deciduous broadleaf forest (6000, 2000 and 700 km^2^, respectively; see Fig. S4 within the online supplementary material). Major changes observed in the Indian peninsula are related to cropland change [1]. Changes in soil and vegetation water content are highly time correlated (R^2^ = 0.52) in cropland areas, whose surface area is relatively stable, and is likely related to development in irrigation systems, as reported elsewhere [1,52].

The East Coast of Australia has suffered an increase in wildfires due to climate change [60–62] and an increase in deforestation due to land clearing policy change [58,59]. This region is susceptible to climate variability induced by the Pacific and Indian Oceans [60] and is very sensitive to wildfires [61]. Furthermore, after the 2008 global financial crisis, many countries became laxer with environmental conservation policies to allow the exploitation of natural resources, as in the case of Australia [58,59]. As we show in Fig. 2E, both soil and vegetation water content have suffered a heavy decrease (5.1% and 7.2% per year, respectively) over the last decade, followed by a decrease in NDVI and annual cumulative rainfall (2.4% and 5.2% per year, respectively). This is also reflected in the observed land cover change, where evergreen forest and grassland lost 29 000 and 20 000 km^2^, respectively, and shrubland areas increased by 40 000 km^2^ due to wildfires and land clearing. In this region, vegetation water content is spatially correlated with NDVI (R^2^ = 0.65) and temporally correlated with soil moisture (R^2^ = 0.79), which reflects the fact that the areas with vegetation water content loss and vegetation degradation have the same evolution in time.

## DISCUSSION AND CONCLUSION

Many studies have suggested that dry regions are getting drier and wet regions are getting wetter. Quantitative assessment of univariate time series of moisture and drought indices has allowed assessing the spatial distribution of the change over time and the impacts on vegetation covers. However, no study until now has approached the problem by considering soil and vegetation jointly. We studied soil and vegetation water content changes over the last decade and uncovered clear patterns and clusters of impacts on terrestrial ecosystems. Using SM and VOD together allowed us to disentangle global terrestrial ecosystems’ causes of change (sensitivity) from natural and anthropogenic factors. Clear hotspots of joint SM and VOD trends stand out and can be semantically attributed to five clear categories. We uncover regions affected by deforestation (Amazon, Central Africa), wildfires (East Australia), artificial reforestation (southeast China), abandonment of farm fields (Central Russia, Central Argentina, North America, India) and climate shifts related to changes in precipitation variability (East Africa, North America, Central Russia). From the detected changes in the bivariate plane, we analyse the changes by case and biome and quantify the affected area and the speed of change in the last decade, 2010–20. Despite the limited spatial resolution and period considered here, we show that satellite observations can quantify and routinely attribute environmental responses to human activity. The study also showed contrasting behaviour by identifying statistically significant clusters where some ‘wet regions get drier’, and other ‘dry regions get wetter’. Investigating joint trends of SM and VOD using a co-clustering approach and allowing for smaller clusters is a matter of future research. Such an exercise could be helpful to sharpen the identification and quantification of terrestrial ecosystem changes, also covering those occurring at smaller spatial scales that are not covered here (e.g. deforestation and wildfires in Indonesia, degradation and forest disturbances). Also, the availability of longer time records of L-band microwave satellite data, as secured by future planned missions such as the Copernicus Microwave Imaging Radiometer, as well as advanced approaches to enhance the spatial resolution of the data, could potentially allow for a finer semantic classification of the clusters into better-resolved combinations of natural and anthropogenic factors.

## METHODS

We computed the trends over SM and VOD using the methodology in [13], which exploits the standard Mann-Kendall (MK) statistical trend test [76, 77] to estimate a monotonic trend from arbitrary time series. The method is applied independently for each grid point. Since a strong temporal autocorrelation is present, we account for an AR(1) correction following [13], drastically reducing false-positive rates. We first estimate the temporal autocorrelation at lag *t* − 1, *r*_*t* − 1_, for each grid cell and compute the residual as $y_t^{\prime }= y_t - r_{t-1}y_{t-1}$. Then the MK trend test is performed on this new time series $y_t^{\prime }$. To correct for multiple hypothesis testing, the method applies a permutation method based on clustering, such that a threshold for overall significance is defined on the number of adjacent (neighbouring) significant grid cells and estimated through permutation, where the significance threshold was set to 0.95 up to the randomly distributed cluster. The result allows us to preserve the spatial correlation of the data and minimise the probability of false positives [13]. To obtain merged regions, the test was performed using different quantiles (0.1, 0.25, 0.5, 0.75, 0.9) from the annual distribution of each pixel. The output is a map with well-delimited regions by quantile and variable (see Table 2). Extracted maps from the variable's quantiles are merged to obtain the studied cluster (see the online supplementary material).

**Table 2.** SM and VOD probability distribution change for each region. Quantiles are calculated from the annual distribution and spatial average.

		SM (m^3^·m^−3^)					VOD (standard units)				
		*Q*_10_	*Q*_25_	*Q*_50_	*Q*_75_	*Q*_90_	*Q*_10_	*Q*_25_	*Q*_50_	*Q*_75_	*Q*_90_
North America (north)	2011	0.06	0.09	0.15	0.21	0.27	0.07	0.11	0.15	0.22	0.33
	2019	0.10	0.15	0.21	0.26	0.31	0.07	0.10	0.15	0.22	0.34
North America (south)	2011	0.02	0.04	0.07	0.16	0.25	0.06	0.10	0.17	0.28	0.48
	2019	0.04	0.06	0.12	0.22	0.30	0.09	0.13	0.20	0.31	0.50
Amazon (north)	2011	0.03	0.05	0.11	0.18	0.23	0.14	0.20	0.26	0.32	0.41
	2019	0.02	0.04	0.08	0.14	0.19	0.13	0.19	0.25	0.30	0.37
Amazon (south)	2011	0.06	0.11	0.18	0.24	0.29	0.38	0.50	0.67	0.89	1.01
	2019	0.05	0.10	0.16	0.21	0.26	0.36	0.46	0.61	0.83	0.97
Argentina	2011	0.12	0.16	0.21	0.26	0.32	0.40	0.68	0.95	1.04	1.08
	2019	0.11	0.16	0.22	0.30	0.39	0.37	0.63	0.89	1.00	1.04
East Africa	2011	0.04	0.05	0.07	0.09	0.16	0.03	0.06	0.11	0.17	0.24
	2019	0.05	0.07	0.09	0.14	0.25	0.07	0.11	0.16	0.23	0.31
India	2011	0.08	0.13	0.21	0.34	0.48	0.01	0.08	0.17	0.26	0.39
	2019	0.07	0.12	0.21	0.35	0.52	0.07	0.13	0.21	0.30	0.42
Central Russia (east)	2011	0.04	0.07	0.13	0.19	0.25	0.02	0.06	0.12	0.22	0.40
	2019	0.07	0.11	0.17	0.25	0.36	0.06	0.10	0.16	0.26	0.42
Central Russia (west)	2011	0.04	0.08	0.13	0.18	0.24	0.02	0.06	0.12	0.23	0.38
	2019	0.06	0.10	0.15	0.21	0.30	0.05	0.10	0.17	0.29	0.43
Southeast China	2011	0.20	0.31	0.47	0.65	0.84	0.30	0.39	0.49	0.57	0.63
	2019	0.13	0.19	0.27	0.35	0.43	0.39	0.48	0.57	0.65	0.70
East Australia	2011	0.05	0.09	0.14	0.20	0.26	0.10	0.14	0.23	0.35	0.56
	2019	0.01	0.03	0.06	0.11	0.18	0.02	0.05	0.12	0.24	0.44

### Reproducibility: software and data availability

All the analysis was performed in matlab^®^. Data and code snippets are provided for reproducibility and can be found at https://github.com/IPL-UV/SM-VOD-Trends.

### Data

Soil moisture is an essential climate variable closely related to other relevant land climate variables and atmospheric fluxes such as surface temperature, evapotranspiration and precipitation. SM and VOD are linked to the water and energy cycles over land and are important drivers of ecosystem variability. Quantifying and understanding the spatio-temporal distribution of global SM and VOD and their changes is crucial for hydrological, ecological and climate models.

We use global SM and VOD maps from the ESA’s SMOS mission for 2010–20. Since its launch in November 2009, SMOS is providing multiangular and full polarimetric L-band brightness temperatures that enable the estimation of global maps of the Earth’s surface SM moisture (top 5 cm) every three days with a spatial resolution of ∼25 km and a target accuracy of 0.04 m^3^·m^−3^, and of VOD, which is the degree of attenuation of microwaves through the vegetation, and is used here as a proxy for vegetation water content and biomass. The SMOS INRA-CESBIO (SMOS-IC) algorithm was designed by INRA (Institut National de la Recherche Agronomique) and CESBIO (Centre d’Etudes Spatiales de la Biosphère) to perform global retrievals of SM and L-VOD. SMOS-IC is based on the two-parameter inversion of the L-band Microwave Emission of the Biosphere model as defined in [24,78] and considers the pixel homogeneous. The SMOS-IC version 2 SM and VOD products provided in the 25-km EASEv2 grid and in NetCDF format are used here [24].

The NDVI from MODIS and rainfall from the precipitation estimation from remotely sensed information using artificial neural networks–climate data record (PERSIANN-CDR) were used as ancillary data for this study. The terra moderate resolution imaging spectroradiometer (MODIS) vegetation indices (MOD13Q1) version 6 data are generated every 16 days at 250-m spatial resolution as a level 3 product. It provides the NDVI choosing the best available pixel value from all the acquisitions within the 16 days. The rainfall dataset comes from PERSIANN-CDR. The precipitation estimation comes from remotely sensed information using artificial neural networks–climate data record (PERSIANN-CDR) version 2.2 monthly global rainfall data with a spatial resolution of 0.25° between 60S–60N latitudes. The precipitation estimate is produced using the PERSIANN algorithm on GridSat-B1 infrared satellite data, and the artificial neural network training is done using the National Centers for Environmental Prediction stage IV hourly precipitation data.

The MODIS also provides yearly global land cover types (2001–20) at 500-m spatial resolution (MCD12Q1), including six different classification schemes. The MCD12Q1 collection 6 product was derived using supervised machine learning methods with MODIS surface reflectances from the terra and aqua platforms for improved classification accuracy [79]. For this work, we have considered the annual plant functional types classification scheme (type 5), which divides the terrestrial surface into eleven broad categories: water bodies, evergreen needle leaf forest, evergreen broadleaf forest, deciduous needle leaf forest, deciduous broadleaf forest, grasslands, shrub lands, cereal croplands, broadleaf croplands, urban and built-up lands, permanent snow/ice and barren or sparsely vegetated. The MCD12Q1 was then spatially degraded to a 30-km spatial resolution. However, instead of providing only dominant land-cover-type values for the broader 30-km grid cells to track land cover changes, we computed annual fractional land cover maps to analyse land cover changes for the study period (2010–20). We calculated these maps using the Google Earth Engine cloud platform [80], considering 500-m pixel size categories within each broader grid cell (30 km).

All data were harmonised to the common spatial scale of 25 km and monthly temporal resolution from June 2010 to July 2020. The generated data are available from Zenodo at https://doi.org/10.5281/zenodo.7660170.

## Supplementary Material

nwad026_Supplemental_File
