# Supplementary material for: Soil and vegetation water content identify the main terrestrial ecosystem changes
Source: Natl Sci Rev. 2023 Feb 7;10(5):nwad026. doi: 10.1093/nsr/nwad026 (PMC10089587; doi:10.1093/nsr/nwad026)
Supplement: nwad026_Supplemental_File [file nwad026_supplemental_file.pdf]

Supplementary Information for

Soil and vegetation water content identify the main terrestrial ecosystem changes

Diego Bueso, Maria Piles, Gustau Camps-Valls, Philippe Ciais, Jean Pierre Wigneron, Álvaro Moreno-Martínez

S.1: Identified clusters by quantile and variable

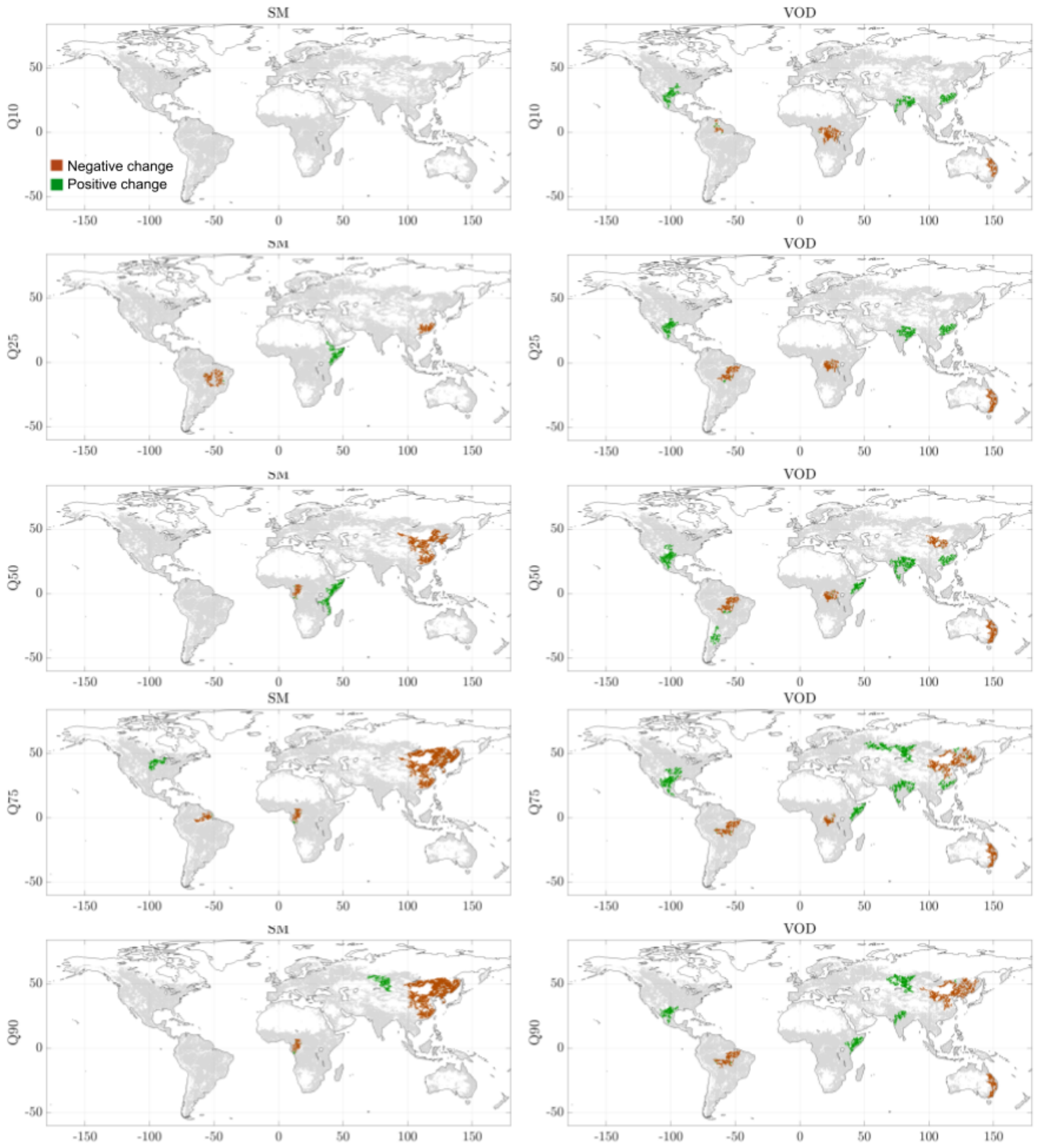

Figure 1: Detected sensitive regions to SM and VOD changes by quantile from annual distribution. The northeast China coast cluster was removed from the study due to potential interference in microwave data.

S.2: Identified clusters by variable

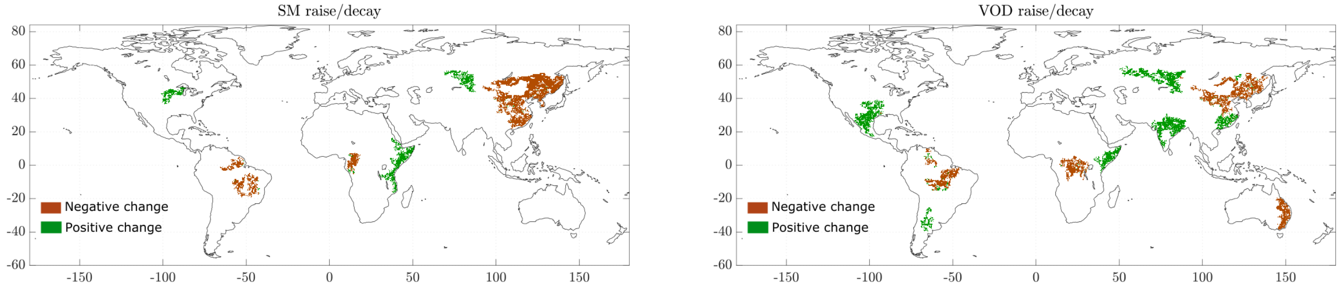

Figure 2: Detected sensitive regions to SM (left) and VOD (right) changes. The raise (decay) patterns are shown in green (brown). The northeast China coast cluster was removed from the study due to potential interference in microwave data.

S.3: Cross-relation between variables

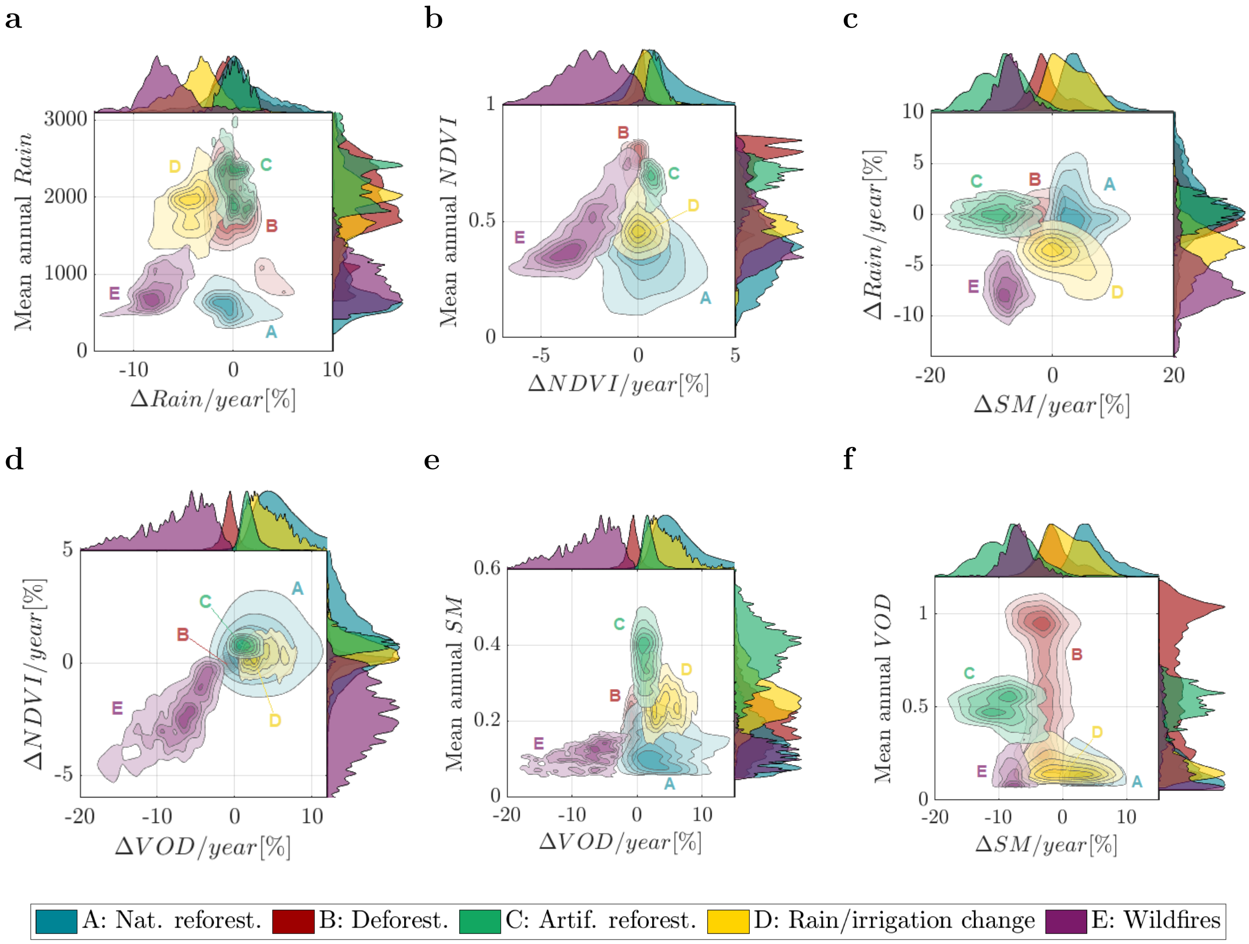

Figure 3: Estimated joint and marginal distributions of (a) trends in rainfall and mean annual rainfall, (b) trends in NDVI and mean annual NDVI, (c) trends of SM and rainfall, (d) trends of VOD and NDVI, (e) trends in VOD and mean annual SM, and (f) trends in SM and mean annual VOD, for the identified clusters. The distinct sensitivity to rainfall and trend in VOD of clusters A and D, related to natural reforestation and rain shift or irrigation change, respectively, is evidenced in (a), (c) and (e). Areas with highest mean annual values of VOD related to high biomass are the most sensitive to changes in SM (f). The sensitivity in NDVI and VOD are highly correlated (d), being the exception Cluster D related to rain shift or irrigation change regions with little change in NDVI for the study period (b).

Table 1 shows the separability score S between the different clusters (A-E) depending on the different combinations of variable trends (ΔSM, ΔVOD, and ΔNDVI). The score S is a standard measure of cluster separability that accounts for intercluster Mahalanobis distances; the lower the value, the most separable the cluster is. We observe that the optimal pair combination is obtained with (ΔSM,ΔVOD). This combination of variables maximizes cluster separability in all cases and yields the best overall results (lowest OS).

Table 1: Cluster evaluation metrics (intercluster separability score) with different input combinations for each considered cluster (A-E), and the overall separability measure, OS.

Variable combination	Cluster					
	A	B	C	D	E	OS
(ΔSM)	0.10	0.12	0.13	0.08	0.10	0.11
(ΔVOD)	0.10	0.11	0.17	0.15	0.06	0.12
(ΔNDVI)	0.15	0.12	0.20	0.20	0.06	0.14
(ΔSM,ΔVOD)	**0.07**	**0.08**	**0.10**	**0.06**	**0.05**	**0.07**
(ΔSM,ΔNDVI)	**0.07**	**0.08**	**0.10**	0.07	**0.05**	0.08
(ΔVOD,ΔNDVI)	0.08	0.08	0.13	0.12	**0.04**	0.09

S.4: Land cover change

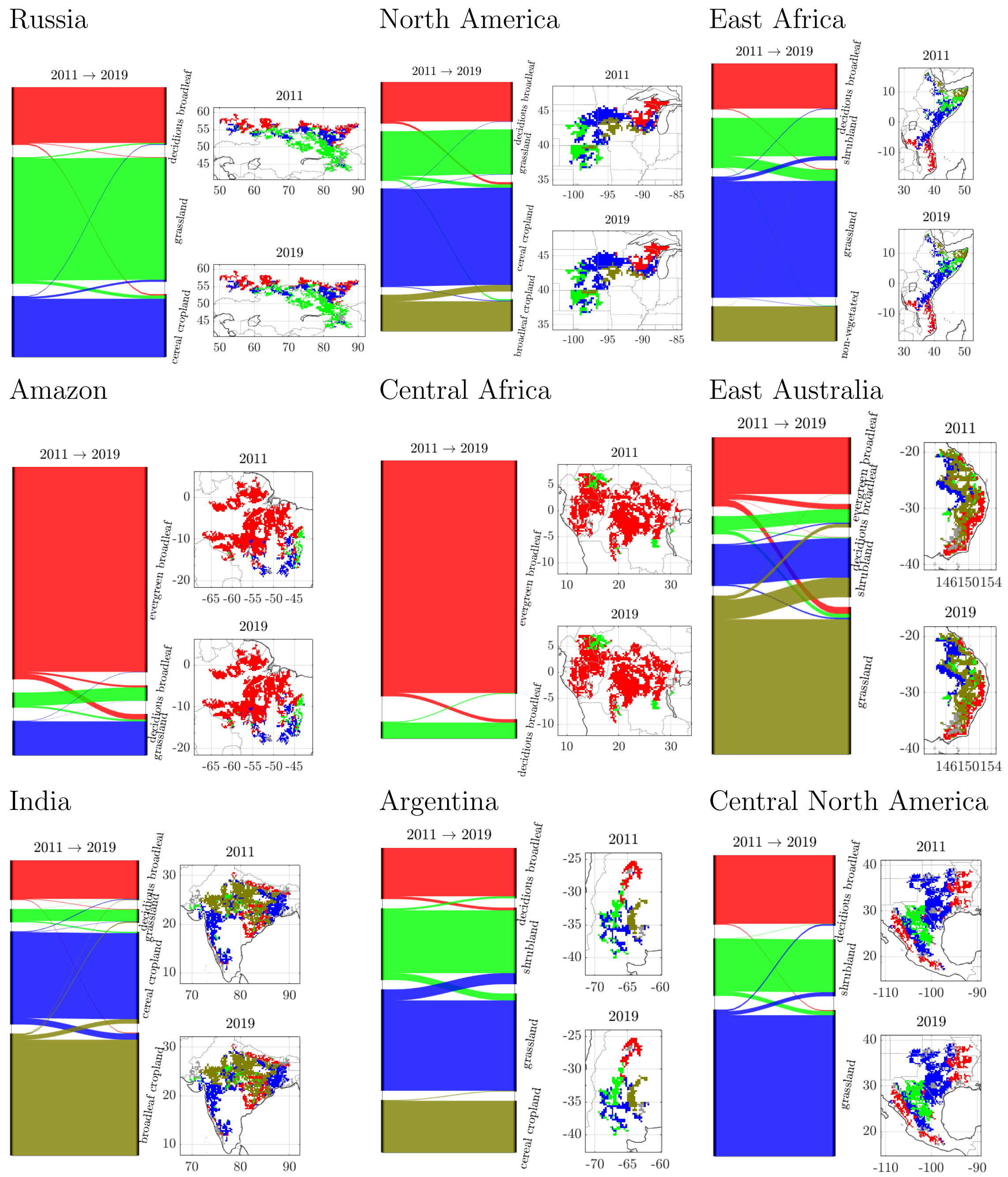

Figure 4: Main changes of land cover between 2011 and 2019 over the regions contained in the identified Clusters: Cluster A related to natural reforestation (Russia, North America, East Africa), Cluster B related to deforestation (Amazon, Central Africa), Cluster E related to wildfires (East Australia), Cluster D related to rain shift or irrigation change (India, Argentina, Russia and Central North America). The Southeast china region and Cluster C related to artificial reforestation is not showed because no major land cover changes have been observed (see also Fig.2).

S.5: SMOS-IC L-VOD and soil moisture products

The simultaneous retrieval of SM and VOD is based on theoretical analyses showing the possibility of decoupling the effects of SM and VOD and performing 2-Parameters (SM and VOD) retrievals from multi-angular and dual-polarized SMOS L-band observations [1]. These theoretical analyses have been validated from evaluations of both the SM and VOD products. A review of these analyses has been made in the SMOS-IC reference paper [2], and we provide here the key elements of the review. SMOS-IC SM has been evaluated against SM model output simulations at a global scale and in situ observations from the ISMN networks (International Soil Moisture Network, Dorigo21). Results have shown the high quality of the SMOS-IC SM, which ranked first of all SMOS products in many intercomparison analyses [3, 4]. Similarly, the SMOS-IC L-VOD retrievals have been evaluated in numerous studies. As a direct evaluation is not possible (there is no large-scale product of vegetation water content VWC (kg/m2)), we used several proxies Li20. Assuming the average moisture content of vegetation is relatively constant at the interannual scale, L-VOD is closely related to vegetation biomass [5]. For instance, L-VOD is strongly spatially correlated ($R > 0.8$) to biomass and vegetation height (a proxy of biomass) at continental and global scales using reference biomass maps [2, 6]. Interannual variations (IAV) in L-VOD have also been shown to be strongly correlated to the IAV of key factors controlling the IAV of biomass in several forest ecosystems, such as the IAV in forest fraction in the Brazilian Amazon (where forest fraction can be used to monitor deforestation intensity, Qin et al., 2021, Li et al., 2022), and the IAV in burnt areas in the Siberian forests [7] and Australia [8].

Regarding inputs, the SMOS-IC SM and VOD retrievals use very few auxiliary inputs. Contrary to some other retrieval algorithms, SMOS-IC is well known as it does not use any vegetation (as NDVI or LAI) or hydrological variables as inputs, making the independent application studies much more robust. SMOS-IC only uses soil and vegetation temperature parameters estimated from ERA5 model simulations. A detailed analysis of the uncertainties associated with L-VOD retrievals has been made by Fan19. Based on a bootstrap cross-validation method, the analysis considered internal errors (associated with the algorithm process and noise on the SMOS TB observations) and external errors (associated with the reference aboveground biomass (AGB) maps used to calibrate the L-VOD / AGB relationships). Fan et al. showed that external errors are the dominant term in the uncertainties associated with L-VOD. There is an order of magnitude between uncertainties arising from internal and external errors. Considering combined internal and external errors, the relative uncertainties associated with the AGC stocks and changes in the AGC stocks over the tropics are 20-30%. Similar orders of magnitude were found at continental scales.

S.6: Qualitative comparison with previous literature

Many studies have been published trying to characterise the changes in terrestrial ecosystems using trend analysis over various drivers. A direct intercomparison to these studies is challenging, and this is not even possible for many reasons. Here we analyse the works closely related to ours [9, 10, 11, 12, 13, 14]. Still, crucial differences were observed that preclude a direct comparison because

- *different considered variables*, as none of these works considers SM, and only [12] and [13] consider VOD, but such VOD was retrieved at high frequencies (mostly X-band), which is very sensitive to strong saturation effects in dense vegetation [15]
- *different study periods*, as only [13, 14] have an (unfortunately short) coincident period of a few years only;
- *different and single variables*, as changes have been typically identified using a single variable (mainly LAI or NDVI), and the results are assessed by comparison with others-
- *adopted methodology to estimate trends* is typically different. For example, except for [11], the rest of the methods consider non-monotonic trends and do not care for the spatial homogeneity of clusters. We use the method proposed in [11], so a key multiple hypothesis testing correction is applied, which reduces the amount of false positive trends (estimated by as much as 25%, cf. [11]). These properties of the applied approach impact the relevance and statistical significance of the identified groups and consequently make a direct comparison more convoluted.
- *spatial resolution* used is generally higher[9, 11, 14] than the 0.25º used in our work, and in the most similar case, i.e. [12], the study period is completely different. This may have an impact when analysing defragmented cropland areas like in Europe.
- *issues related to data preprocessing*, such as the RFI contamination in mainland China that may affect some L-band estimates and that had to be screened out in our study.

Still, an incomplete (yet reasonable) comparison is possible only with the works [9, 11, 13], essentially because (a) some similar period but at a higher spatial resolution of LAI trends is studied in [9], which allows us to clarify some divergent patterns, (b) we use the same methodology for computing trends as in [11] over a similar period, but on SM and VOD instead of LAI, and (c) only [13] considers VOD (yet retrieved from high-frequency observations and strongly affected by saturation issues in dense vegetation) at similar spatial and temporal resolutions over a similar period to estimate trends in global terrestrial ecosystems, but using standard linear regression without a non-monotonic or statistical significance test. All these points have been analyzed to compare the methodologie and findings, as well as similarities and contrasting patterns found in the literature, and are reported and summarized in Table 2.

Reference	Comparable?	Study period	Data & methods	Similar observations	Different or contrasting observations
[1] Chen et al.	**YES,** similar methodology and study period, but using LAI only	**2000–2017**	* LAI (MODIS)<br><br>* 0.05º<br><br>* Signif. Trends (Mann–Kendall, P ≤ 0.1)	We also observe significant changes in Indian crops and capture patterns of natural reforestation in western China. Other croplands, like the dry (southern) US corn belt, are also identified, probably due to irrigation shifts.	We identified the patterns in northeast China (see S2). Still, We decided to remove this region from the study due to potential RFI not being captured by the RFI flags provided and impacting SMOS brightness temperatures and hence the SM and VOD products.<br><br>We capture patterns of natural reforestation in southeast China. As in Chen's Fig.2, our work also highlights the southeast China region as being the most active in reforestation.<br><br>In Sahel, we identify deforestation, but Chen et al. clearly state a greening cluster compared to previous studies using AVHRR (2000–2016, similar period); the sub-Saharan cluster is missing as in our work.<br><br>We did not find statistically significant clustered trends due to croplands in Europe, probably due to the highly heterogeneous (defragmented) croplands, which cannot be captured at the SMOS resolution, or the lack of saturation of L-VOD, unlike in vegetation indices.
[12] Zhu et al.	**NO,** completely different period and methodology	**1982–2009**	* LAI products (GIMMS, GLOBmaps, GLASS)<br>* 0.5º		
[13] Cortés et al.	**YES,** the same method and overlapping period, but using LAI only	**2000–2018**	* LAI products (AVHRR, MODIS, Cyclops)<br><br>* 0.05º<br><br>* Multiple hypothesis testing of significant trends	In our study, cereals are associated with clusters A (w/ shrubs) and D (w/ deciduous forests): these are in India and parts of the corn belt.<br><br>A coincident region in the western Siberian plain / central Siberian plateau of Eurasian boreal forests compared to the BU AVHRR LAI in [13]-Fig.1.	Some greening patterns are missing in our results (Europe, SouthEast China) when looking at the LAI trends extracted from the BU AVHRR LAI in [13]-Fig.1. Not identifying croplands in Europe is due to our coarser spatial resolution. Irrigation management change can be detected in India, where only the VOD has a significant positive trend. Our analysis only considers interannual changes; seasonal phenology is not covered here.<br><br>Northeast China has been removed from the study due to potential RFI impacting SMOS measurements in the region<br><br>We identify east Africa as natural reforestation in cluster A, as in [17,39-41], but [13] Cortes et al. do not.
[14] Andela et al.	**NO,** completely different period and methodology	**1988–2008**	* NDVI<br><br>* VOD *0.25º<br><br>* monthly		
[17] Zhang et al.	**YES,** different method but same VOD data over the slightly overlapping period	**1992-2012**	* LAImax (GIMMS3g, MODIS)<br><br>* NDVI<br><br>* bi-monthly<br><br>* 1/12º<br><br>* VOD, 0.25˚<br><br>* linear regression, non-monotonic or significance tests	Non-conclusive pattern in Amazonia; [17] shows mainly a 'not-significant' correlation between LAI and rainfall trends in Amazonia. A decline in the woody cover is observed for high-rainfall zones (>1800 mm/yr), presumably caused by deforestation. This is also confirmed by (Brandt et al., 2017), who identified woody cover changes between 1992–2011 in approximately half of sub-Saharan Africa and attributed it to human population growth and the related deforestation practices, as we capture in cluster B.	
[20] Munier et al.	**NO,** different period and completely different resolution	**1999–2015**	* LAI (MODIS)<br><br>* 0.05º<br><br>* Significant Trends		
**Our work**		**2010-2020**	*** SM**<br><br>*** VOD 0.25º monthly**<br><br>***same method as [13]**		

Table 2: Comparison to related approaches in the literature.

References

[1] Wigneron JP, Waldteufel P, Chanzy A *et al.* Two-dimensional microwave interferometer retrieval capabilities over land surfaces (smos mission). *Remote Sens* 2000; **73**: 270–282.

[2] Wigneron JP, Li X, Frappart F *et al.* Smos-ic data record of soil moisture and l-vod: historical development, applications and perspectives, remote sens. *Env.* 2021; **254**: 8.

[3] Al-Yaari A, Wigneron JP, Dorigo W *et al.* Assessment and inter-comparison of recently developed/reprocessed microwave satellite soil moisture products using ismn ground-based measurements. *Remote Sensing of Environment* 2019; **224**: 289–303.

[4] Ma H, Zeng J, Chen N *et al.* Satellite surface soil moisture from smap, smos, amsr2 and esa cci: A comprehensive assessment using global ground-based observations. *Remote Sens* 2019; **231**: 11121.

[5] Frappart F, Wigneron JP, Li X *et al.* Global monitoring of the vegetation dynamics from the vegetation optical depth (vod): a review. *Remote Sensing* 2020; **12**: 2915.

[6] Li X and al WJP. The first global soil moisture and vegetation optical depth product retrieved from fused SMOS and SMAP L-band observations. *Remote Sensing of Environment* 2022; **282**: 113272.

[7] Fan L, Wigneron JP, Ciais P *et al.* Siberian carbon sink reduced by forest disturbances. *Nat. Geosci.* 2022; .

[8] Qin Y, Xiao X, J-P* W *et al.* Carbon loss from forest degradation exceeds that from deforestation in the brazilian amazon. *Nature Climate Change* 2021; **11**: 442–448.

[9] Chen C, Park T, Wang X *et al.* China and india lead in greening of the world through land-use management. *Nature Sustainability* 2019; **2**: 122–129.

[10] Zhu Z, Piao S, Myneni RB *et al.* Greening of the Earth and its drivers. *Nature Climate Change* 2016; **6**: 791–795.

[11] Cortés J, Mahecha MD, Reichstein M *et al.* Where are global vegetation greening and browning trends significant? *Geophysical Research Letters* 2021; **48**: e2020GL091496.

[12] Andela N, Liu YY, van Dijk AIJM *et al.* Global changes in dryland vegetation dynamics (1988-2008) assessed by satellite remote sensing: comparing a new passive microwave vegetation density record with reflective greenness data. *Biogeosciences* 2013; **10**: 6657–6676.

[13] Zhang W, Brandt M, Peñuelas J *et al.* Ecosystem structural changes controlled by altered rainfall climatology in tropical savannas. *Nature Communications* 2019; **10**: 671.

[14] Munier S, Carrer D, Planque C *et al.* Satellite leaf area index: Global scale analysis of the tendencies per vegetation type over the last 17 years. *Remote Sensing* 2018; **10**.

[15] Li X, Wigneron JP, Frappart F *et al.* Global-scale assessment and inter-comparison of recently developed/reprocessed microwave satellite vegetation optical depth products. *Remote Sensing of Environment* 2021; **253**: 112208.
